# Density and strength distribution of the subchondral bone plate of the canine talus

**DOI:** 10.3389/fvets.2025.1679334

**Published:** 2025-10-28

**Authors:** Yasamin Vali, Magdalena Müller-Gerbl, Henri van Bree, Walter Dingemanse, Ingrid Gielen

**Affiliations:** ^1^Department of Small Animals and Horses, University of Veterinary Medicine, Vienna, Austria; ^2^Institute of Anatomy, Department of Biomedicine, Musculoskeletal Research, University of Basel, Basel, Switzerland; ^3^VetMedImage, Erondegem, Belgium; ^4^Kennel and Paddock Veterinary Rehabilitation and Hydrotherapy, James Lane, Grazeley Green, Berkshire, United Kingdom; ^5^Department of Morphology, Imaging, Orthopedics, Rehabilitation and Nutrition, Faculty of Veterinary Medicine, Ghent University, Merelbeke, Belgium

**Keywords:** canine (dog), densitometry, talus, computed tomography, bone strength, bone density

## Abstract

**Introduction:**

The subchondral bone plate plays a critical role in load transfer across joints. Its density distribution reflects the joint’s loading history, and variations in density are likely associated with changes in the material properties of the subchondral bone. This study aimed to evaluate the mechanical strength of the subchondral bone plate of the canine talus and correlate it with the subchondral bone density.

**Methods:**

Twenty paired cadaveric tali from large breed dogs were included in the present study. Test points were selected and marked on the subchondral bone plate, where mechanical strength was assessed using indentation testing to record the maximum penetration force. The density at these test points was measured using computed tomographic osteoabsorptiometry (CT-OAM).

**Results:**

The result revealed that density and strength of the subchondral bone plate were not uniformly distributed across its surface. A strong correlation was observed between subchondral bone density and mechanical strength across all specimens, with the areas of highest density corresponding to the areas of greatest mechanical strength.

**Discussion:**

The present study’s key finding—a strong correlation between subchondral bone density and mechanical strength—highlights the potential of using subchondral bone density as a reliable indicator of mechanical strength. This relationship offers important insights for clinical assessments and research on joint biomechanics. Furthermore, the use of CT-OAM provides a non-invasive method to evaluate both the density and mechanical strength of the subchondral bone plate, enabling valuable longitudinal studies on subchondral bone properties in dogs.

## 1 Introduction

The importance of bones in vertebrates is foundational to both anatomical and physiological knowledge, making it a crucial focus for research. Bones not only provide a firm skeleton that grants strength, stability, and movement to the organism, but they also encase and protect vital organs. Additionally, bones play significant roles in hematopoiesis and homeostasis, serving as reservoirs for essential minerals (1). The skeleton effectively supports the body’s movement and is continuously subjected to various forces. According to Wolff’s law, bone density adapts and is influenced by mechanical forces, suggesting a dynamic relationship between mechanical stress and bone remodeling (2). This concept is encapsulated in the “Mechanicostat” theory, which links the degree of strain induced by mechanical forces to skeletal remodeling (3, 4). This phenomenon not only impacts long bones but also affects cuboid intra-articular bones, as joints mediate different degrees of forces and consequently undergo strains and remodeling (5).

The important aspects about the Mechanicostat and Wolff’s law is that they play a role in pathology as well as in physiology. If the biomechanics of the joint alter, it leads to changes in the loading distribution and, consequently, changes in the subchondral bone’s density distribution ((6, 7)). Understanding this process allows us to evaluate joint loading retrospectively by assessing subchondral bone density.

Computed tomography (CT) is a diagnostic imaging method that creates cross-sectional two-dimensional (2D) images and three-dimensional (3D) reconstructions by analysis the absorption and attenuation of X-ray photons in tissue (8). CT is widely used in clinical practice to assess fracture risk by analyzing changes in bone structure, specifically the cortical and trabecular bone. Computed Tomography Osteoabsorptiometry (CT-OAM), developed by Müller-Gerbl et al. (7), processes CT images to create topographic maps of bone tissue density (7). By use of CTOAM relative density differences in joints can be evaluated, highlighting the subchondral bone’s changes in consequence of absorbing mechanical forces and supporting articular cartilage, which remodels faster than the cartilage itself in response to mechanical changes (9).

Based on the aforementioned knowledge in the field of bone density and CT-OAM, the current study is designed to evaluate the correlation between the density distribution of the canine talus, as assessed by CT-OAM, and the penetration strength of the talar subchondral bone plate.

## 2 Materials and methods

### 2.1 Specimens

Twenty paired canine cadaver tali were included in this study, sourced from the Department of Morphology at the Faculty of Veterinary Medicine, Ghent University, Belgium (Table 1). The bones came from various large breed dogs, all weighing over 25 kg, to ensure a sufficiently large testing surface for indentation testing. Prior to the testing, the bones were stored frozen until the day before the tests. Exclusion criteria included any macroscopic cartilage damage, degenerative changes, or signs of osteoarthrosis observed in the CT images before testing.

**Table 1:** Summary of the clinical data of the specimens and correlations between the measured density values and the mechanical strength for each specimen.

Specimen	Breed	Gender	Age (years)	Side	*r*^2^
SBD-1 SBD-2	German Shepherd	Male	2	Left Right	0.83 0.86
SBD-3 SBD-4	Belgian Shepherd (Malinois)	Female	5	Left Right	0.93 0.91
SBD-5 SBD-6	American Bulldog	Female	N/A	Left Right	0.94 0.89
SBD-7 SBD-8	German Shepherd	Female	4	Left Right	0.96 0.94
SBD-9 SBD-10	Bernese Mountain Dog	Male	6	Left Right	0.95 0.91
SBD-11 SBD-12	Mixed Breed	Male	8	Left Right	0.83 0.88
SBD-13 SBD-14	Labrador Retriever	Female	4	Left Right	0.78 0.84
SBD-15 SBD-16	Great Dane	Female	7	Left Right	0.94 0.92
SBD-17 SBD-18	Poodle	Male	5	Left Right	0.79 0.82
SBD-19 SBD-20	Mixed Breed	Male	N/A	Left Right	0.89 0.93

*r*^2^, coefficient of determination; N/A, not available.

**Figure 1:**
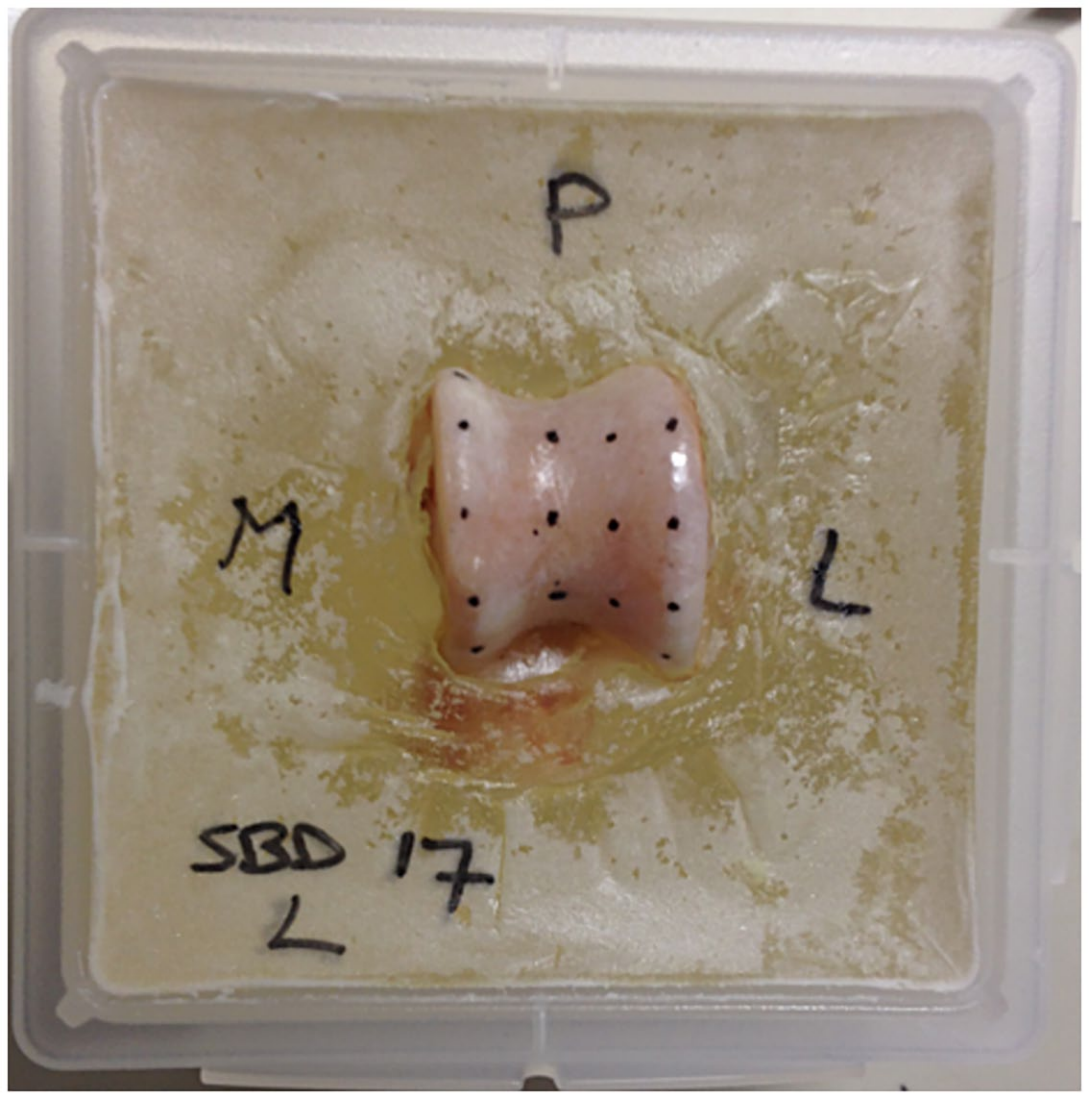
Specimen prepared for testing. The talus is embedded in polymethylmethacrylate (PMMA), and the testing grid is marked on the surface of the subchondral bone. The letters P, M, and L indicate posterior, medial, and lateral, respectively, to provide clear orientation for the location of the various testing points.

Based on the size of the specimen, three or four rows of test points were marked on the articular surface (Figure 1). The spacing between these points was measured using a caliper, ensuring a minimum distance of 7 mm between them to prevent interference during testing. The test points were positioned on the medial and lateral trochlear ridges, as well as in the trochlea tali. In certain cases, a fourth row was added on the sloped medial surface of the lateral trochlear ridge.

### 2.2 Image acquisition and analysis

Image data was acquired using a 16-slice CT scanner (Somatom Sensation, Siemens, Erlangen, Germany). Scans were conducted both before and after indentation testing to verify full penetration of the subchondral bone plate and to accurately register the indentation points for comparison with the CTOAM images. The CT images were exported in DICOM format to commercially available software (Analyze 11.0, Biomedical Imaging Resource, Mayo Foundation, Rochester, MN, USA), used to complete the CT-OAM workflow, as it is previously reported by the same authors (10). Initially, the talus was segmented using the software’s segmentation algorithm. The subchondral bone plate of the articulating surface was isolated and reconstructed. The maximum bone density was projected onto the articular surface using a maximum intensity projection (MIP). In this step, the 3D data volume (in voxels) of the subchondral bone plate is converted into a 2D image (in pixels), with each pixel representing the maximum value based on Hounsfield Units (HU). This value is obtained from the voxels along a line perpendicular to each pixel in the 2D image. The length of this line, or the MIP depth, was determined by the thickness of the subchondral bone plate and was set to 6 different reconstrcutions ranged from 5 to 8 mm to visualize all the test points.

For quantification purposes, the density values (in HU) were converted to 8-bit values, creating 256 density values, which were then evenly distributed into eight bits according to the literature (11). Each bit contained 32 density values. A density maximum was defined as an area with density values in the two highest density bins of the densitogram. To compare individual subchondral bone density distributions, a 30 × 30 unit grid was superimposed on the densitogram of both the proximal and dorsal views of the trochlear ridges. The grid edges were positioned to ensure the entire joint surface fit within. The number of units in each grid remained constant to standardize the coordinates of the density maxima. The x- and y-coordinates were used to describe the location of the density maxima on the joint surface.

### 2.3 Indentation testing

All ligaments and other soft tissue structures were removed from the talus, and the articular cartilage covering the subchondral bone plate was manually excised using a scalpel blade positioned nearly parallel to the bone surface to avoid damaging the subchondral bone. Before testing, the talus was secured in polymethylmethacrylate (PMMA) to aid positioning during the tests (Figure 1). The PMMA blocks were then fixed in a custom-made screw-on frame with a ball joint, allowing the indentation needle to be positioned perpendicularly to the surface. The ball joint was locked in place during testing.

**Figure 2:**
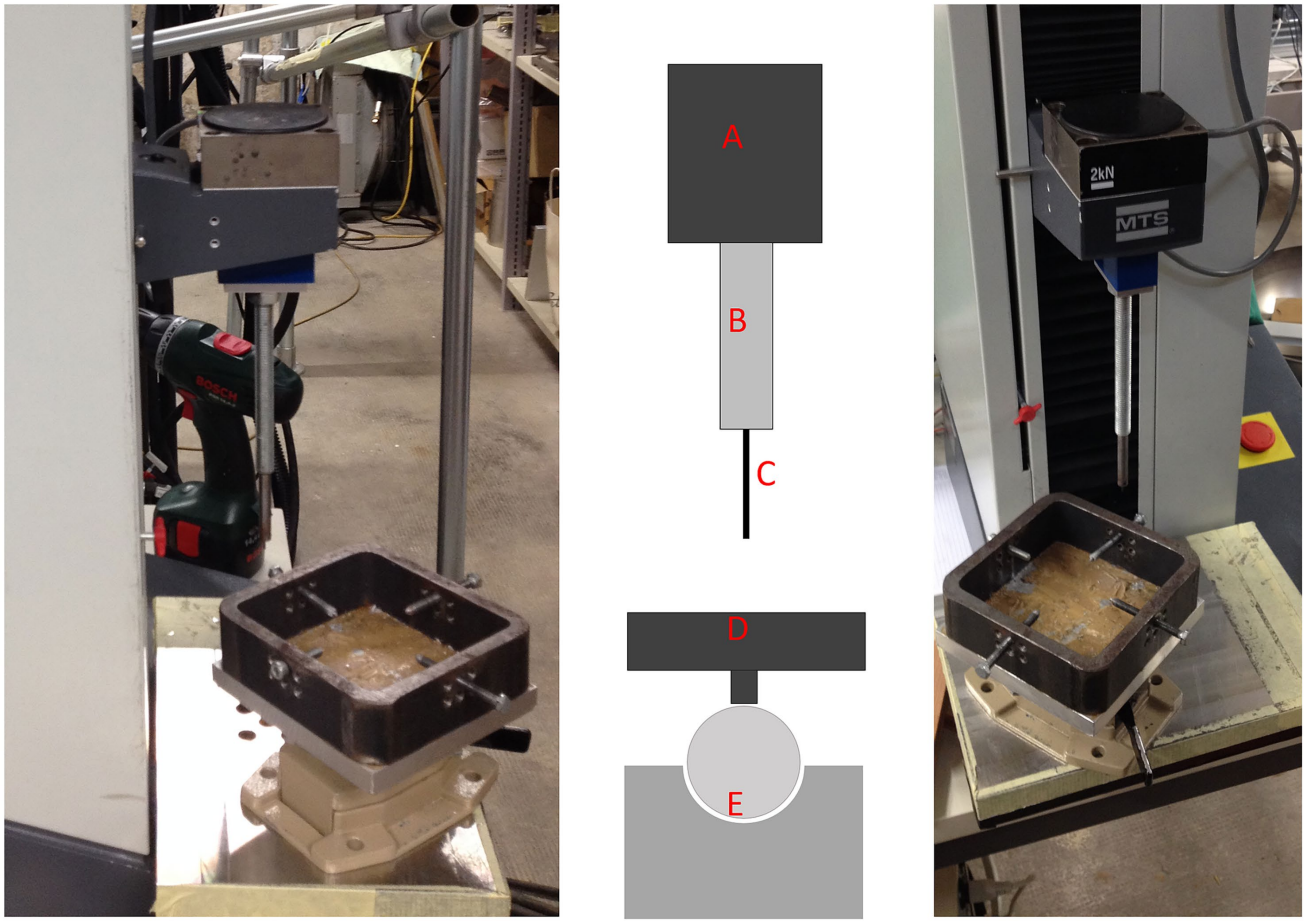
Overview of the materials testing machine used in this study. Left: lateral view; middle: schematic view; right: frontal view. The schematic view highlights the different components of the testing setup: A, Force registration module; B, testing needle attachment; C, testing needle; D, positioning table for the PMMA blocks, which can be secured with 4 screws; E, ball-bearing joint for optimal positioning of the needle perpendicular to the surface of the subchondral bone plate.

**Figure 3:**
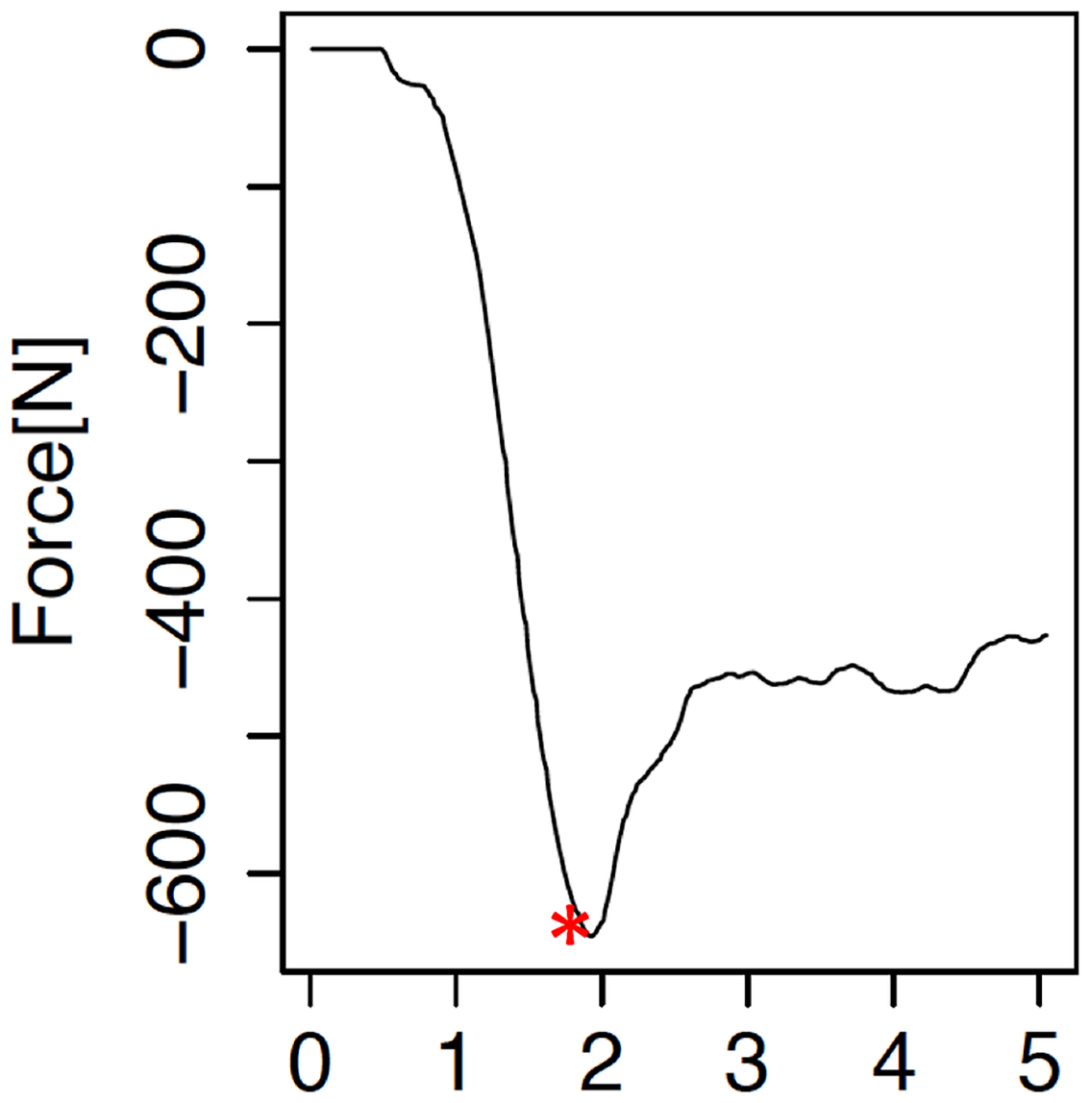
Force-time curve resulting from each testing point. The curve illustrates the increase in resistant force (in Newtons, N), reaching a maximum (*), at which point the subchondral bone plate is penetrated, and the needle enters the trabecular bone.

Indentation testing was conducted using a material testing machine (Synergie 100, MTS Systems, Eden Prairie, MN) with custom software that measured the reactive force of the indentation needle in Newtons (N) at a constant speed of 1 mm/s (Figure 2). A steel needle, 2 mm in diameter, was used to create a standardized indentation at each test point (Figures 1, 2). This process generated a time-force curve for each test point, showing the increase in resistance until maximum force was reached and the needle fully penetrated the subchondral bone plate (Figure 3). The maximum force, or penetration force, was recorded in Newtons and entered into a standardized grid.

**Figure 4:**
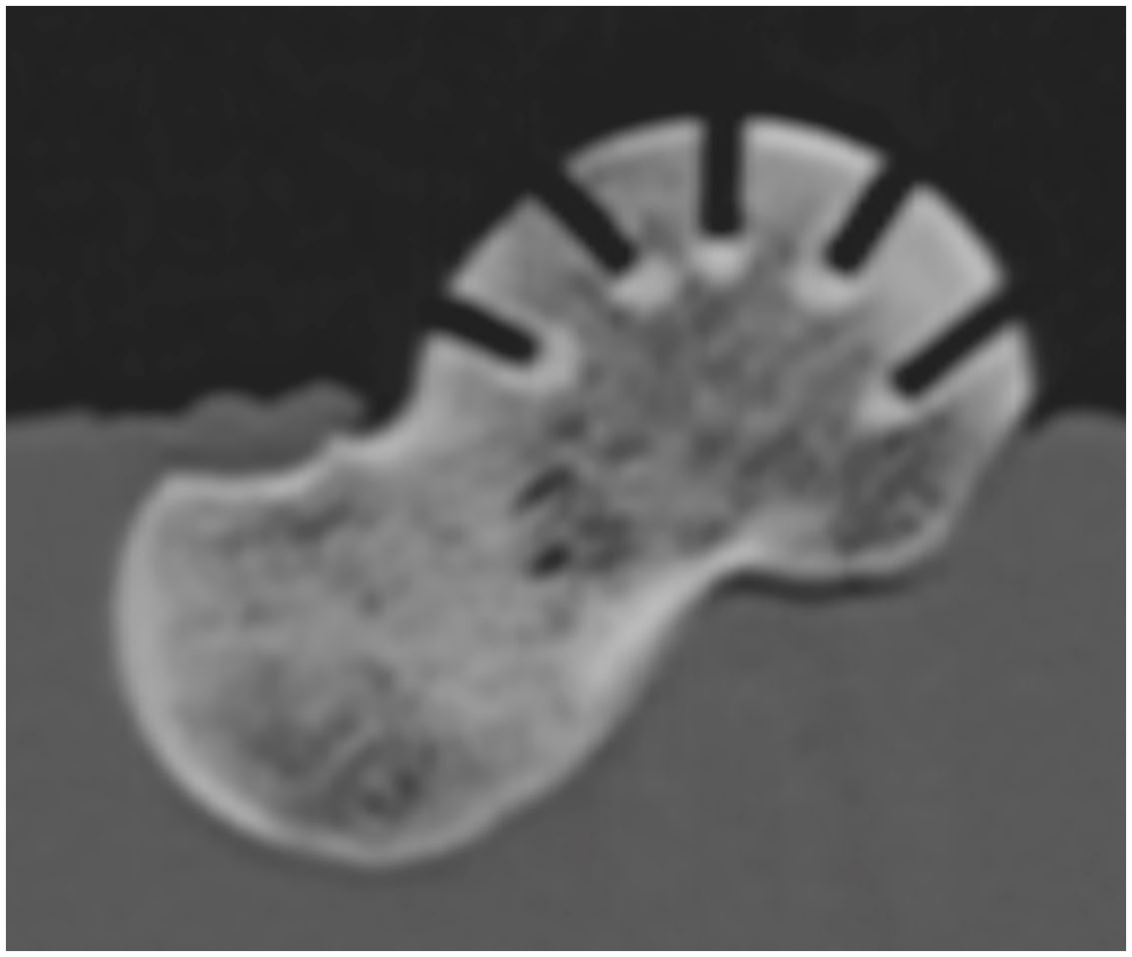
Sagittal reformatted CT image of a talus after testing, demonstrating the perpendicular orientation of the needle hole and the full penetration of the subchondral bone plate.

After the indentation testing, a second set of CT images was acquired (Figure 4). The density at each indentation point was measured using pre-testing CTOAM images, with post-testing CTOAM images ensuring accurate positioning of the region of interest (ROI). The correlation between measured mechanical strength and subchondral bone density was analyzed using linear regression, and the Pearson product–moment correlation coefficient was calculated for all the tested tali.

## 3 Results

**Figure 5:**
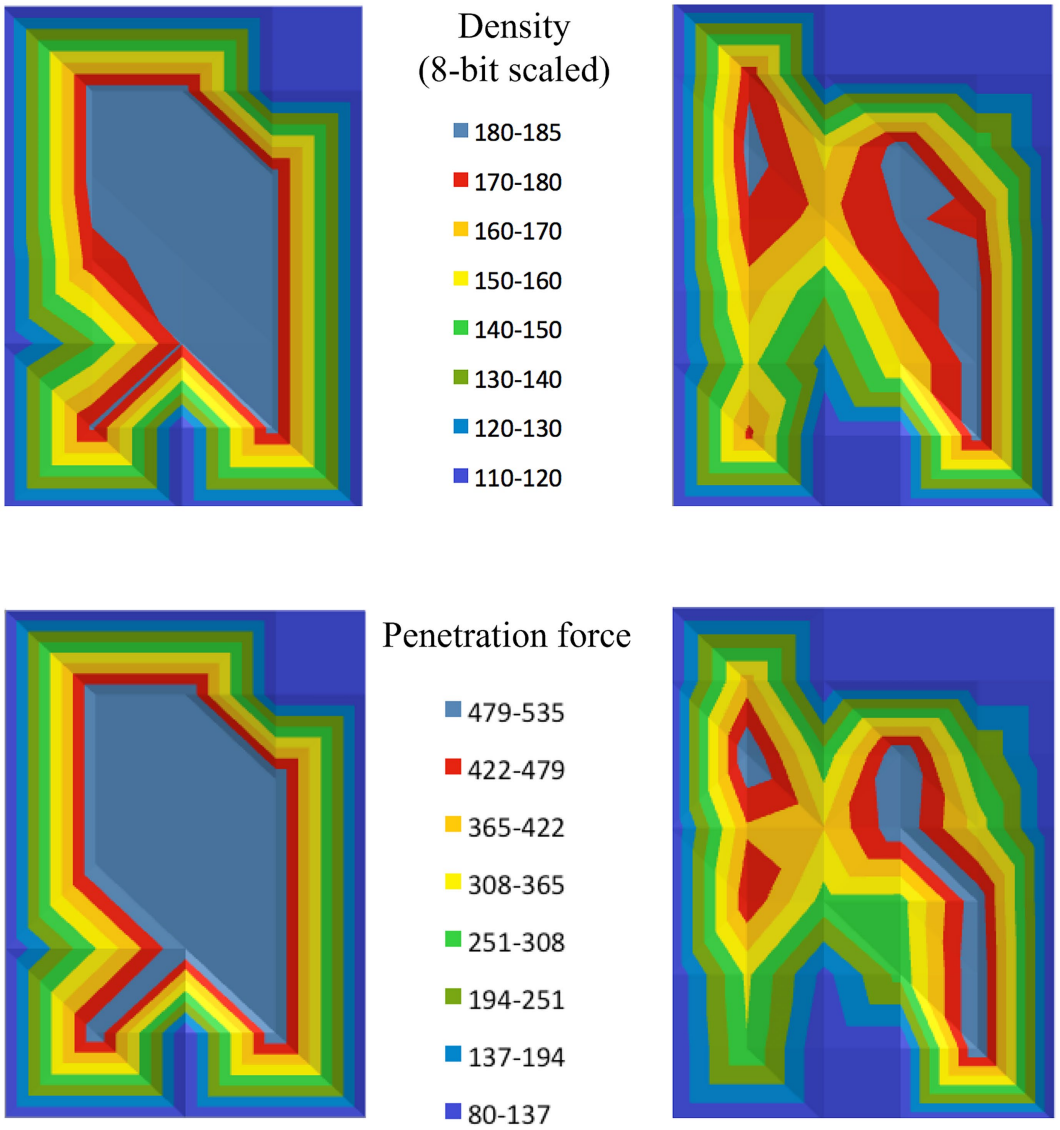
Density and strength distribution results for the talus of two different dogs (one on the left, and one on the right). The top row displays the density distribution, while the bottom row shows the strength distribution, visually representing the penetration force at each testing point.

The subchondral bone density distribution across the canine tali was found to be non-homogeneous, with one or more distinct areas of density maxima observed in each specimen. These maxima indicate regions where the bone density was significantly higher compared to the surrounding areas, suggesting variability in the loading patterns on the joint surface. Additionally, the pattern of subchondral bone density distribution varied between individual specimens (Figure 5).

In addition to the density distribution, the mechanical strength of the subchondral bone plate was also found to be unevenly distributed. Similar to the density distribution, the mechanical strength showed regions of maxima, where the bone resisted penetration forces more effectively, indicating stronger areas within the subchondral bone plate (Figure 5).

The distribution pattern of mechanical strength closely mirrored the pattern of bone density distribution revealed by CTOAM. Both density and strength maps displayed interindividual variation, with each talus showing a unique distribution of these properties, reinforcing the link between the bone’s structural characteristics and its mechanical behavior (Figure 5).

Visual comparison of the subchondral bone density distribution and the mechanical strength distribution revealed marked similarities. In all cases, the regions with density maxima corresponded to areas with the highest mechanical strength.

Statistical analysis confirmed that there was no significant difference in the location of density maxima and strength maxima. The *p*-value of 0.512 indicates that the maxima for density and strength consistently co-localized, supporting the hypothesis that bone density is a strong predictor of mechanical strength in the subchondral bone plate.

**Figure 6:**
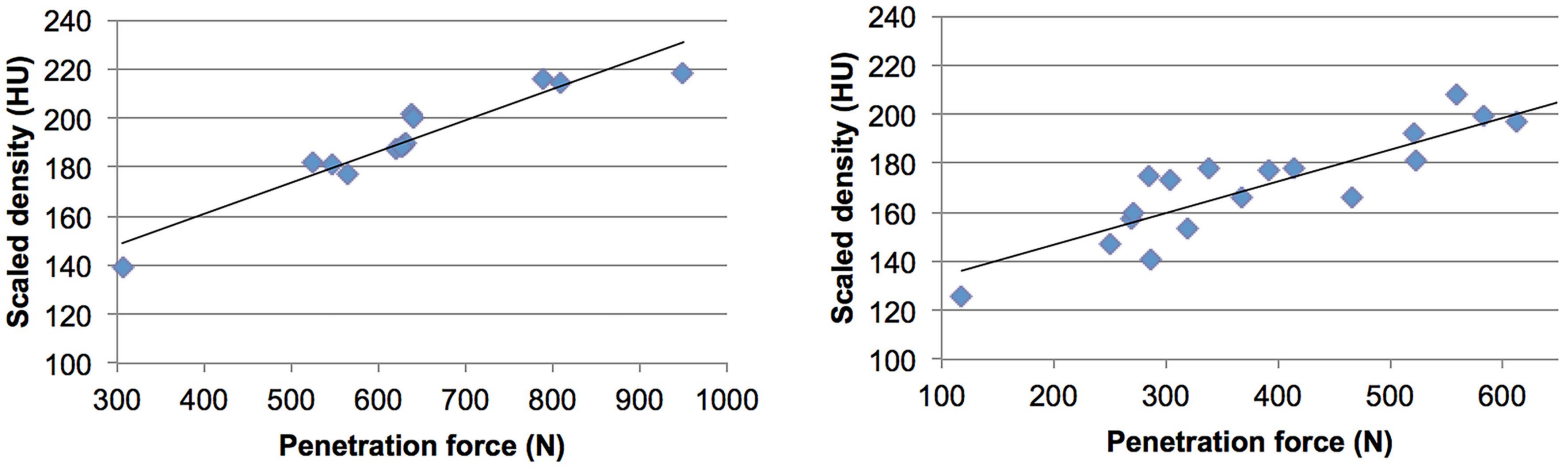
Regression plots showing the correlation between density values (in 8-bit scaled Hounsfield Units) and penetration force (in Newtons) for two dogs described in Figure 5. The left and right plots correspond to the respective dogs.

High correlations were found between the measured density values and the mechanical strength at each test point across all tali. The correlation coefficient (*r*^2^) ranged from 0.78 to 0.96, with a mean value of 0.89, demonstrating a strong and statistically significant relationship between bone density and strength (*p* value < 0.01) (Figure 6; Table 1).

## 4 Discussion

The subchondral bone density and mechanical strength across the canine tali were found to be unevenly distributed, with distinct areas of maxima observed in each specimen. These maxima, where the bone density and strength were significantly higher, suggest variability in joint surface loading patterns. The distribution patterns of density and strength closely mirrored each other, showing interindividual variation, and consistently co-localized. Statistical analysis confirmed a strong and significant correlation between bone density and mechanical strength, reinforcing the idea that bone density is a reliable predictor of mechanical strength in the subchondral bone plate.

Evolutionarily and physiologically, the body compensates for varying mechanical demands on the skeletal system through a process known as bone remodeling (5). This adaptive mechanism, governed by Wolff’s law, suggests that bone density and structure are constantly adjusted in response to the mechanical forces exerted upon them (2). When a particular area of the skeleton experiences increased load or stress, osteoblasts are activated to lay down new bone tissue, enhancing the bone’s density and strength in that region (2, 5). Conversely, in areas of reduced mechanical demand, osteoclasts resorb bone tissue, preventing unnecessary metabolic costs (5). This dynamic process not only optimizes the skeleton’s structural integrity, making it more resilient to fractures and wear, but also ensures that the body efficiently allocates resources. Over evolutionary time, this ability to adapt has contributed to the survival of species by enabling them to withstand different environmental challenges, maintain mobility, and protect vital organs, all while minimizing the risk of injury (5).

This study investigates the correlation between subchondral bone density and the mechanical strength of the subchondral bone plate in the canine talus. The results revealed that subchondral bone density is not uniformly distributed, exhibiting local variations and significant interindividual differences. Previous studies on canine subchondral bone density, particularly in the Labrador Retriever talus (10) and mixed breeds’ elbow joints (12), have shown similar variations. The observed interindividual variation in this study can likely be attributed to the inclusion of different large breed dogs, each with unique joint loading patterns and distribution, leading to differences in subchondral bone density.

In humans, similar patterns of subchondral bone density distribution have been documented within the same joint, with these density patterns closely linked to strength distributions in various joints, such as the wrist, ankle, patella, and shoulder (13–16). The high correlation between bone density and strength observed in these studies is mirrored in the results of this study on dogs, suggesting that CTOAM can be effectively used to assess the mechanical strength of the subchondral bone plate in canines as well.

The mechanical strength of the subchondral bone plate is crucial in the (patho)physiology of cartilage and subchondral bone. Studies on horses, for example, have shown significant correlations between bone mineral density, elastic modulus, and energy to failure, with these parameters varying according to anatomical location within the joint (17). Changes in bone density can affect these functional parameters and may contribute to the development of joint pathologies such as osteoarthrosis (OA) and osteochondrosis (OC) (18, 19). In Labrador Retrievers, the locations of density maxima in the tarsocrural joint align with the typical sites of OC lesions (10). Although the exact etiology of OC remains under debate in both humans and dogs, microtrauma or repetitive biomechanical stress is likely to play a significant role.

These biomechanical factors influence subchondral bone density, and increases in density may impair the regenerative and reparative potential of the subchondral bone and overlying cartilage. While further research is needed to fully understand the role of biomechanical loading in the development of OC lesions in dogs, current evidence supports the hypothesis that joint loading plays a critical role in canine OC (18, 19). Additionally, a mismatch between the biomechanical characteristics of the subchondral bone and articular cartilage may contribute to the initiation or propagation of orthopedic pathologies, including OC and OA, particularly when density changes occur in the subchondral bone plate (18, 19).

Overall, these findings suggest that subchondral bone density, as measured by CTOAM, is a reliable indicator of mechanical strength. The consistent correlation across all specimens supports the use of density measurements as a non-invasive method to predict the mechanical properties of the subchondral bone plate in canine joints.

## Data Availability

The raw data supporting the conclusions of this article will be made available by the authors, without undue reservation.
